# Identifying high crash risk segments in rural roads using ensemble decision tree-based models

**DOI:** 10.1038/s41598-022-24476-z

**Published:** 2022-11-21

**Authors:** Maryam Iranmanesh, Seyedehsan Seyedabrishami, Sara Moridpour

**Affiliations:** 1 Faculty of Civil and Environmental Engineering, Tarbiat Modares University, Tehran, Iran; 2 Civil and Infrastructure Engineering Discipline, RMIT University, Melbourne, Australia

**Keywords:** Civil engineering, Scientific data

## Abstract

Traffic safety forecast models are mainly used to rank road segments. While existing studies have primarily focused on identifying segments in urban networks, rural networks have received less attention. However, rural networks seem to have a higher risk of severe crashes. This paper aims to analyse traffic crashes on rural roads to identify the influencing factors on the crash frequency and present a framework to develop a spatial–temporal crash risk map to prioritise high-risk segments on different days. The crash data of Khorasan Razavi province is used in this study. Crash frequency data with the temporal resolution of one day and spatial resolution of 1500 m from loop detectors are analysed. Four groups of influential factors, including traffic parameters (e.g. traffic flow, speed, time headway), road characteristics (e.g. road type, number of lanes), weather data (e.g. daily rainfall, snow depth, temperature), and calendar variables (e.g. day of the week, public holidays, month, year) are used for model calibration. Three different decision tree algorithms, including, Decision Tree (DT), Random Forest (RF) and eXtreme Gradient Boosting (XGBoost) have been employed to predict crash frequency. Results show that based on the traditional evaluation measures, the XGBosst is better for the explanation and interpretation of the factors affecting crash frequency, while the RF model is better for detecting trends and forecasting crash frequency. According to the results, the traffic flow rate, road type, year of the crash, and wind speed are the most influencing variables in predicting crash frequency on rural roads. Forecasting the high and medium risk segment-day in the rural network can be essential to the safety management plan. This risk will be sensitive to real traffic data, weather forecasts and road geometric characteristics. Seventy percent of high and medium risk segment-day are predicted for the case study.

## Introduction

More than ninety per cent of crash fatalities occurred in low and middle-income countries between 2013 and 2016, where only half of the world’s registered vehicles are found in those countries^1–3^. The rate of fatal crashes on rural roads is much higher than on urban roads due to the higher average and variance of vehicular speed, the larger proportion of heavy vehicles, lack of access to emergency care^4^ and some other factors that may be different in countries. In Iran, between 2006 and 2019, around 30% of crashes occurred on rural roads (170,060,000 out of 708,747,714 crashes per year occurred on rural roads). However, around 70% of crash fatalities (8,707,143 out of 12,076,500) occurred on rural roads. The crash frequency data in Iran is more accurate compared to the crash severity data. In Iran, the crash frequency data is recorded by the police, and it is accurate. However, several organisations are involved in recording the number of fatalities and injuries, which results in some inaccuracy in crash severity since the crash severity data is sometimes not updated. For instance, the injured person at the scene of the crash may die in the hospital a few days later. Since the police database and the hospital database are not connected, the crash will be classified as an injury crash based on the police data and a fatal crash, according to the hospital data. Thus, the crash severity data is not very accurate and consequently, predicting crash severity and identifying high-risk segments may not be precise, while predicting crash frequency is more accurate.

Safety studies focus on two different aspects, including planning and operation. In the planning aspect of safety studies, the main focus is to determine the causes of crashes, analyse the effects of traffic crashes and identify and prioritise the countermeasures. Meanwhile, the operational aspect of safety studies focuses on traffic operations, providing road users with satisfactory service and accurate risk information. Machine Learning (ML) techniques can be used as a powerful tool in traffic safety operations to assist in analysing the crash data, detecting incidents and predicting crash risk. Accurate analysis of crash data helps detect or predict the factors influencing crashes and potentially saves time and lives^5^.

The first objective of this paper is to identify the influencing factors on crash frequency on two-way rural roads. The potential influencing variables investigated in this research include traffic data (e.g. average and variance of traffic flow and speed, time headway, the proportion of heavy vehicles), road characteristics (e.g. road type, number of lanes, free-flow speed and road capacity), weather data (e.g. average daily rainfall, snow depth, horizontal visibility, daily temperature, sunlight during 24 h, wind speed) and calendar data (e.g. day of the week, public holidays, month, year).

ML techniques have the ability to learn and enhance from experience without being explicitly programmed, and therefore ML techniques are among the most popular prediction techniques in the fourth industrial revolution^6^. Therefore, tree-based models are used in this paper to predict crash frequency on rural roads. Tree-based models are easier to explain and interpret compared to other ML techniques^7^. This paper presents three different tree-based models, including Decision Tree (DT), Random Forest (RF) and eXtreme Gradient Boosting (XGBoost), known as the most interpretable ML techniques. This paper also compares the accuracy of these models in predicting crash frequency and crash risk using the identified influencing factors.

The second objective of this paper is to present a framework to develop a spatial–temporal crash risk map based on the results from crash frequency prediction models. The risk map is developed in GIS to determine the dangerous road segments with high crash frequency potential on various days. The high-resolution crash frequency data is analysed by identifying the number of crashes per day (temporal analysis) and within a 1500-m radius around loop detectors (spatial analysis). The data has been widely spread over rural roads for several years, enabling us to produce a spatial–temporal risk map. The presented risk map helps traffic safety organisations to identify high-risk segments and apply countermeasures to reduce the possibility of crash occurrence and decrease the crash se verity.

This paper will focus on the crash frequency of two-way rural roads with high data resolution of crash data, traffic data, weather data and calendar data that has attracted less attention in the literature. Another contribution of this paper is to develop different tree-based models and employ concordance analysis techniques using traditional evaluating measures for selecting the most accurate prediction model for generating a dynamic crash risk map.

This paper is structured as follows. The literature review is presented in the following section. It is followed by explaining the dataset used in this study in Data Description’ Section. The methodology and the models developed to predict the crash frequency are presented in “Methodology” section. Afterwards, the results are presented, and the findings are discussed in “Results and discussion” Section. The final section presents concluding remarks and highlights directions for future research.

## Literature review

ML algorithms are generally able to learn from experience without being specifically programmed, which is why for developing intelligently analyzed data and real-world applications, machine learning algorithms are important^6^. In comparison to other ML techniques, tree-based models are easier to explain and interpret. The use of Decision Trees (DT) models has been widely reported in road safety literature^8–21^. Tree-based models are used in different areas of road safety, such as crash severity analysing and prediction, predicting the crash occurrence, and crash frequency analysis and prediction. This paper focuses on analysing and predicting crash frequency on rural roads.

A Decision Tree (DT) is a useful algorithm used to analyse crash frequency as non-parametric algorithms that do not consider previous mathematical assumptions of relationships between variables involved. Chen and Wang (2009) developed DT learning models for Automatic Incident Detection (AID) in their research. In testing its performance, simulated data is used, including traffic flow parameters such as volume, occupancy, time headway and vehicle speed at both upstream and downstream detectors. This DT produced a satisfactory incident detection rate, with an acceptable false alarm rate and mean detection time. Decision tree learning can be more effective than neural networks when using discretised data^14,21^.

A random forest (RF) -developed by Breiman^22^- is a collection of numerous superimposed decision trees that are developed through a selection and validation process. RF models have been used in different crash frequency studies, some of which will be mentioned. Siddiqui et al.^23^ applied RF to identify and examine significant variables associated with total crashes and severe crashes per traffic analysis zone (TAZ) in four counties of the state of Florida. The variables that were further verified and supported by the RF results and which carried a higher weight of importance for total crashes per TAZ were: the total number of intersections per TAZ, airport trip productions, light truck productions, and total roadway segment length with 35 mph posted speed limit^23^.

The Extreme Gradient Boosting (XGBoost) method was initially proposed by Chen and Guestrin in 2016, which generally produces high accuracy and fast processing time while being computationally less complex and cost-effective^24^. XGBoost has already been used by Meng^25^ to predict the occurrence of crashes based on a number of data sources, including road geometric design and historical crash data, as well as traffic and weather data. Parsa et al. use XGBoost to detect the occurrence of crashes using a set of real-time data comprised of traffic factors [average speed/volume 5 min after crash/non-crash at upstream /downstream, difference of speed/volume between upstream and downstream and Average daily traffic of link (ADT)], network characteristics (number of lanes, distance from Central Business District (CBD) and connectivity), demographic characteristics (gross residential density (house unit/acre) on unprotected land), land use characteristics (Area percentage of the buffer zone around link covered by commercial land use), and weather features (Ordinal feature from 1 for sunny to 4 for stormy weather conditions). The results of the study showed that XGBoost detects crashes with high accuracy, a high detection rate, and a low false alarm rate of 99%, 79%, and 16%, respectively^16^.

Schlögl et al.^26^ employed and compared a series of statistical learning techniques with respect to their predictive performance and discussed the importance of determining factors of crash occurrence from the ensemble of models. They considered a binary classification task, with the dichotomous target variable indicating events (crashes) and non-events (no crashes). Findings substantiate that a trade-off between accuracy and sensitivity is inherent to imbalanced classification problems. Results show the satisfying performance of tree-based methods such as RF and XGBoost models, which exhibit accuracies between 75 and 90% while exhibiting sensitivities between 30 and 50%. This study uses a dataset that has a high temporal (1 h) and spatial (250 m) resolution covering the entire highway network of Austria for four consecutive years, inclusive of 45 variables related to road condition, geometry, traffic, weather, and crash time^26^.

Table 1 presents a summary of recent studies on crash analysis. These studies often used a fusion of two or more databases such as crash data, traffic data, weather data, and road geometry data. Those studies mostly used ML techniques such as XGBoost and RF to predict crash frequency^15,16,18,27–33^. In the current studies, some factors are less seen, such as traffic volume hourly and daily, time headway in traffic data, free-flow speed, road capacity in road geometric data, snow depth, sunlight during 24 h in weather data and public holidays, month and year in calendar data.

**Table 1:** Recent studies on crashes analysis and prediction.

Authors (year)	City/country	Model	Input	Output
Peijie Wu et al.^27^	North Carolina (urban and rural)	Poisson/ NB^1^ model, HP/HNB^2^model, RIHP/RIHNB^3^ model	Crash and geometric data	Crash frequency
Das, Geedipally and Fitzpatrick^28^	Ohio, Washington (rural)	Multiplication model based regression analysis	Crash, traffic, weather and geometric data	Annual crash frequencies at segments
Roland et al.^29^	Chattanooga (urban)	MLP^4^	Crash and weather data	Accident detection
Wen et al.^18^	Texas (urban and rural)	LightGBM^5^, SHAP^6^ Model, MARS, CART, AdaBoost, NN, XGBoost	Crash, traffic and geometric data	Total crash, ROR/RE crash occurrence per segment per year
Ramírez and Valencia^30^	Bogota (Urban)	LGCP^7^, MCMC^8^	Crash, traffic and calendar data	Daily frequency and severity
Zhang, Waller and Jiang^31^	California (urban and rural)	RF, ERTs^9^, AdaB, GTB^10^	Crash, traffic and geometric data	Crash frequency
Peng et al.^32^	Shanghai (urban)	LR^11^, RF, MLP	Crash and traffic data	Accident detection
Schlögl^15^	Austria (rural)	RF, XGBoost	Crash, traffic, weather and geometric data	Accident detection
Afghari, Haque and Washington^33^	Queensland, Australia (rural and urban)	NB, OL^12^	Crash, traffic and geometric data	Crash count and crash severity
Parsa et al.^16^	Chicago (urban)	XGBoost	Crash, traffic, weather, geometric, demographic and land use data	Accident detection

^1^Negative binomial.

^2^Poisson hurdle/Negative binomial hurdle.

^3^Random intercepts Poisson hurdle/ random intercepts negative binomial hurdle.

^4^A multilayer perceptron.

^5^Light gradient boosting machine.

^6^Shapley Additive exPlanations.

^7^Log Gaussian Cox process.

^8^Monte Carlo Markov Chain.

^9^Extremely randomized trees.

^10^Gradient tree boosting.

^11^Logestic regression.

^12^Ordered logit.

In this paper, using a fusion of crash, traffic, weather and geometric design databases, the safety status is predicted based on the data of the frequency of crashes in the daily time and within a radius of 1500 m of traffic counters. This study used traffic volume hourly and daily, time headway and average speed and standard deviation in speeds in traffic data, type of road, number of lanes, free-flow speed, road capacity in road geometric data, air temperature, visibility, rainfall, wind speed, snow depth, sunlight during 24 h in weather data and weekday, public holidays, month and year in calendar data. In this study, tree-based classification models such as DT, RF and XGBoost are less applied for forecasting crash frequency are employed to forecast crash frequency to rank high-risk segments in terms of crash frequency. Based on this prediction, a risk map that is a valuable input for safety management is generated.

## Data description

**Figure 1:**
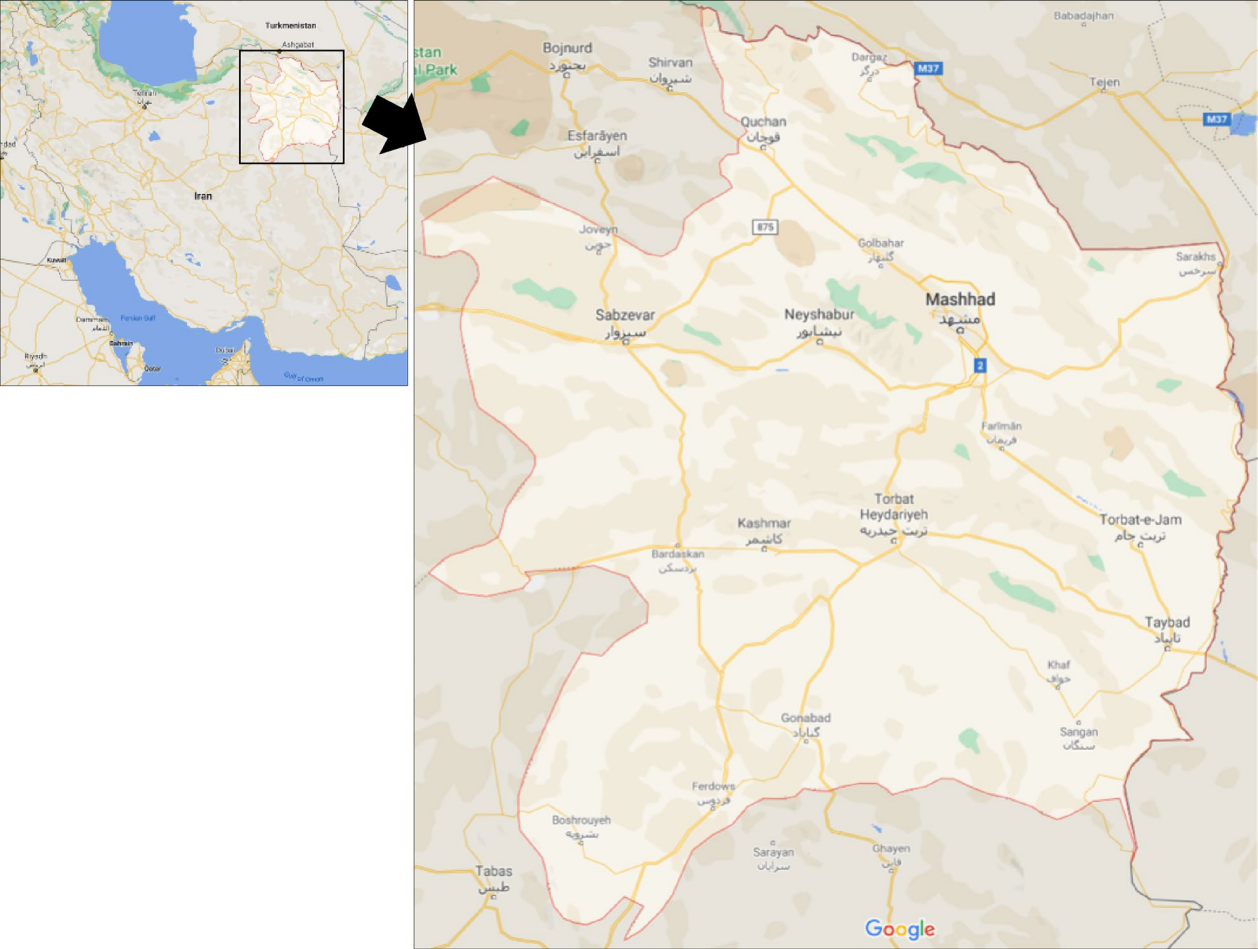
Khorasan Razavi Province and its rural roads map [Maps Data: ©2021 Google].

Iran was included among lower to middle-income economies for 19 years (between 1987 and 2006) and among upper-middle-income economies for 11 years (from 2007 to 2018)^34^. In this research, the rural road network of Khorasan Razavi Province has been used to predict crash frequency on rural roads and develop crash risk maps (Fig. 1). Khorasan Razavi Province is located in the northeast part of Iran and is the second most populated province in Iran. The length of roads under the Ministry of Roads and Urban Development in Khorasan Razavi Province is 5569 km. The seven-year average annual number of vehicles crossing the borders of the province is around 527,000 vehicles^35^.

In this paper, five different data sources, including (1) crash data, (2) traffic data, (3) road geometric data, (4) weather data, and (5) calendar data for a period of seven years from 2014 to 2020, are combined to extract a single dataset for the calibration and validation of the crash frequency models. The five different data sources are explained in the following subsections.

### Crash data

**Figure 2:**
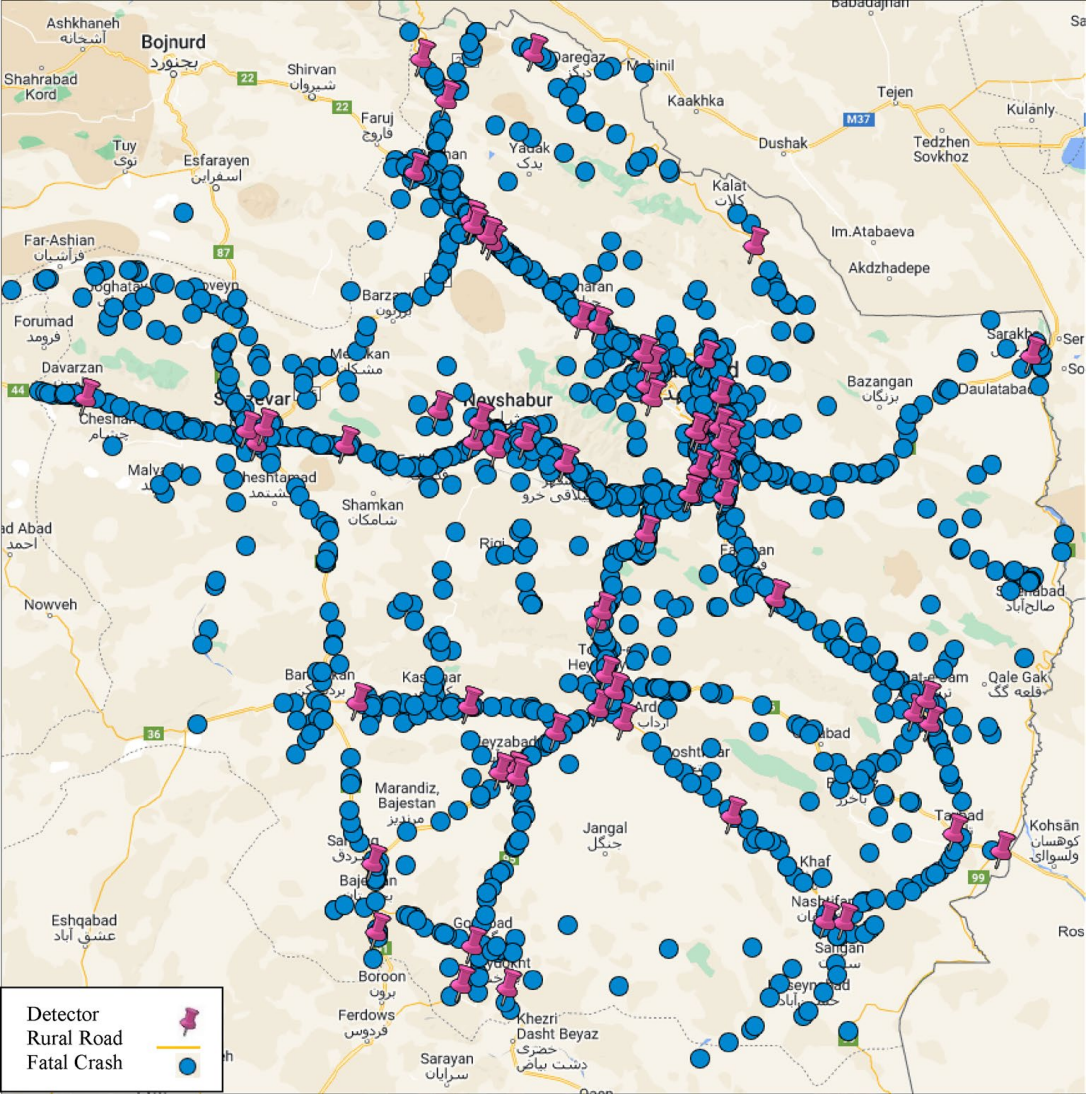
The distribution of crashes and detectors on rural highway network of Khorasan Razavi Province during 2014 to 2020 [Maps Data: ©2022 Google].

Crash data is provided by the Safety Department of Road Maintenance and Transportation of Khorasan Razavi Province. This data is based on police reports comprising 5 different categories of data. The data categories include (1) crash location (e.g. crash ID, city, type of the road, road and segment name, distance from the nearest police station; and latitude and longitude of crash), (2) crash time (e.g. minute, hour, day, month and year), (3) crash severity, including Property Damage Only (PDO), Injury and Fatality), (4) road condition (e.g. lighting, pavement condition, the existence of median, existence of road shoulder, pavement marking, road geometric characteristics, maximum speed), (5) weather data (e.g. daily rainfall, snow depth, temperature). In addition, the details of the vehicle, driver and road users involved in the crash are also considered in the crash data. During the seven-year study period, there were 50,503 crashes reported on the target rural road network. Table 2 presents the number of crashes according to their severity for each year from 2014 to 2020. Figure 2, displays the location of 1668 fatal crashes in this study. As shown in this figure, the data is not limited to a specific road, but it is spread over a rural highway network, including 50,503 crashes and 200 detectors in 5569 kms of road.

**Table 2:** The annual number of crashes for each severity category.

Type crash	Year						
	2014	2015	2016	2017	2018	2019	2020
PDO	2608	2465	2158	2379	2637	4163	3600
Injury	4154	4403	4554	4015	4712	3904	3083
Fatal	279	274	239	207	247	231	191

The crash data has two dimensions, including temporal and spatial. In this study, the temporal dimension is one day that is used to specify time-related variables, and the spatial dimension is a segment that is specified by a circle with a radius of 1500 m around a detector. The 1500-m radius is selected in this research after initial sensitivity analysis. It should be noted that this radius does not cover all network crashes and only covers 80% of crashes.

### Traffic data

One of the main sources of data for crash analysis is traffic parameters obtained from loop detectors. There are 200 loop detectors located in the studied area that record the traffic information for individual vehicles in the form of aggregated data in 5-min time intervals. Figure 2, displays the location of these detectors. For this study, traffic data is provided by the Road Maintenance and Transportation Organization (RMTO)^36^. RMTO has online and historical traffic data from permanent loop detectors, that record vehicle’s volume, speed, headway and type. Since the crash frequency models are calibrated for each day, thus the data aggregated for one-day interval^35^.

The loop detector dataset includes many erroneous and missing values due to detector malfunctioning, which influences the modelling result. Thus, the first step is a data processing to identify false and missing data, and the second is to use data cleaning methods. Data cleaning techniques such as removing irrelevant values, getting rid of duplicate values, converting data types and imputing missing values, which means assuming the approximate value is applied in this paper. After that, traffic-related data is aggregated in 24-h (1 day) intervals since 5-min intervals are too short for capturing crash frequency and in many time intervals of less than 24-h, the frequency of cash is equal to zero, which may largely upset the balance of the data.

Traffic parameters, which are employed in this research, have a high correlation with crash frequency and are consistent with the literature^37^. The traffic data used in this paper include average and variance of traffic flow and speed obtained from one-hour data, average vehicle time headway, and proportion of heavy vehicles.

### Road geometric data

Road geometric data is available from open-source maps such as vector data from the Open Street Map (OSM) and google earth satellite images for each segment. The road geometric characteristics used in this paper include road type, number of lanes, free-flow speed and road capacity determined by the longitude and latitude of each loop^38^. Between 2014 and 2020, there were no changes in the road geometric data.

### Weather data

Another important source of data in this research is weather information. The twenty synoptic meteorological stations of the Iran Meteorological Organization (IRIMO)^39^ collect and archive weather data in the study area. The important weather-related parameters that are available in the weather data include average daily rainfall, snow depth, horizontal view, the amount of cloudiness, average daily temperature, the amount of sunlight during 24 h, duration of the sun’s appearance and wind speed. In addition, weather-related data is aggregated in 24-h (1 day) intervals.

### Calendar data

The year of the crash is an important variable in crash frequency analysis due to safety management plans that may influence the number of crashes each year. In addition, the day of the year usually affects traffic parameters in rural road networks. For instance, the sequence of holidays may increase traffic volume and movement conflicts and consequently increase the frequency of crashes.

### Dataset structure

The crash data for the rural roads of Khorasan Razavi Province combined with traffic data, road geometric data, weather data and calendar data are used to create the dataset for crash frequency model development. Overall, the final dataset includes 252,286 rows, and each row is specified for a day between 2014 and 2020 and a specific segment in this study is called segment-day. There are 42 columns in the dataset, including the number of crashes within the particular day and segment, daily traffic parameters, weather conditions, calendar data, and road geometric characteristics of the segment. All data processing and model development is done in Python 3.8.2.

## Methodology

**Figure 3:**
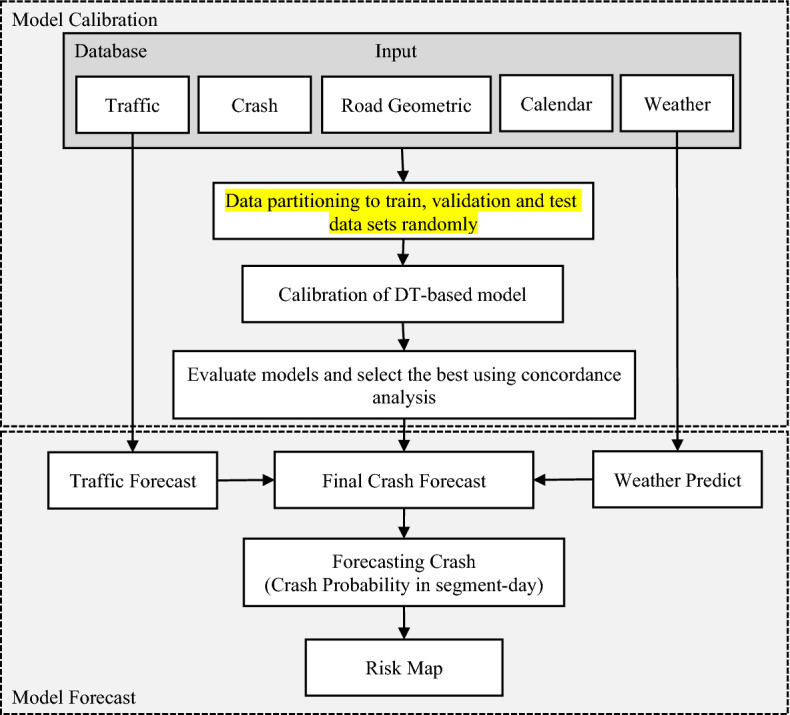
Flowchart for the procedure of generating crash risk map.

The methodology of this paper is described in three subsections: Model input preparation, which includes defining variables and balancing training datasets; model selection, including Decision Tree (DT), Random Forest (RF), and Extreme Gradient Boosting (XGBoost), and model evaluation. Figure 3 shows the algorithm proposed in this research for generating a risk map by utilising multisource data to analyse crash frequency. Model calibration involves joining data and organising a database based on crash data, traffic data, road geometric characteristics, and calendar variables. Step two uses data partitioning to train, validate and test data sets randomly that stratify based on crash frequency. The next step includes calibrating DT-based models, including DT, RF, and XGBoost. The models will forecast the categories of predefined classes from crash frequency that occurred in each segment-day based on real-time or forecasted traffic data, weather, and geometric design. 0-crash, 1-crash, and 2+ crash classes are labelled as low, medium, and high-risk segment-day, respectively.

### Model input preparation

#### Definition of variables

Crash frequency prediction models can be used to identify and rank high-risk segment-day in the rural road network that can be used for safety management plans. To decrease the variance of the dependent variable, crash frequency, the dependent variable is defined as a categorical variable and discrete choice-based analysis is used in this paper. Considering the frequency of crashes in the existing dataset, 98.2 per cent of segment-days have zero crashes, 1.7 per cent have one crash, and 0.1 per cent of segment-days have two or more crashes. Accordingly, the risk-level of the segment-day can be classified into low, medium, and high-risk segment-day. Therefore, if the prediction model identifies the category in which a segment-day will be placed, it will be enough to forecast the risk-level of segment-day in the short-term future.

After employing various types of variables, including discrete and continuous ones, the most significant explanatory variables to develop the crash frequency prediction models are selected and reported in Table 3. Traffic parameters and weather-related variables have better performance and are more significant in terms of feature importance when they are continuous, but calendar variables and road geometric variables have a significant influence when they are assumed as discrete or dummy variables.

**Table 3:** Description of explanatory variables.

Feature	Description	Frequency	Mean	Std
**Crash**				
0	Zero Crash	247,764	–	–
1	One Crash	4,254	–	–
2 +	Two and more crash	268	–	–
**Traffic**				
Flowrate	Flow rate (Vehicle per minute)	–	4.365	5.061
BigCarPercent	Share of heavy vehicle (%)	–	20.427	14.096
VolOnCap	Volume divided by capacity	–	2.380	1.913
SpdOnFrSpd	Speed divided by Free flow speed	–	50.364	44.962
StdspdOnMspd	standard deviation of speed on mean speed	–	0.171	0.275
SpeedOnHeadway	Speed divided by Headway	–	209.635	124.460
**Geometric**				
Road_type	Road type, 2 dummy variables (base: Toll Road) Road_type_1: Main Road = 1, Other:0 Road_type_2: Freeway = 1, Other:0	237,838 252,286	–	–
Line_count	Number of lanes, 3dummy variable (base: 4lane)	–	–	–
**Weather**				
Rain	Amount of rainfall (Millilitre)	**–**	0.522	2.572
Snow	Snow depth (Millimetre)	**–**	5.203	7.526
HorizontalView	Horizontal view length (Meter)	**–**	12,557	3251
Cloudiness	The amount of cloudiness (%)	**–**	2.262	2.378
Temperature	Temperature (Celsius)	**–**	16.320	9.064
AmountSunlight24	The amount of sunlight during 24 h (Millilitre)	**–**	1828	976
LengthSunlight	The length of sunlight per day (Hour)	**–**	6.134	4.944
Wind	Wind Speed (Kilometre per hour)	**–**	8.159	4.509
**Calendar**				
Year	Ordered variable 2014: 0 until 2020:6	–		
Season	Season, 3 dummy variables (base: Winter)	–		
Month	Month, 11 dummy variables (base: March)	–		
Weekday	On week (Saturday to Wednesday) = 1 Other = 0	180,224		
Holiday	Holiday, 1 dummy variable, Holiday = 1 Other = 0	92,272		

#### Balancing the training dataset

Due to the high temporal and spatial resolution of data, the number of segment-day in 0 class (no traffic crashes) is too many compared to the number of segment-day in 1 and 2+ classes. The large number of zero segment-day is highly imbalanced in the dataset. The training dataset comprises 4522 observations with one and more than one crashes in contrast to 247,764 observations with zero crashes, resulting in class imbalance equivalent to an order of 1–55. The imbalanced dataset in which the presence of the majority class overshades the minority classes is the nature of the crash datasets and may result in biased prediction results in the ML method^37,40,41^.

Synthetic Minority Oversampling Technique (SMOTE) is a well-known oversampling technique that was proposed to improve random oversampling^42^. SMOTE is a minority over-sampling technique that generates synthetic samples for the minority class^37^. To overcome the problems of having an imbalanced crash dataset, SMOTE has been applied in this study to create a more balanced training dataset and provide a larger presence to the minority class.

### Model selection

Due to a large number of observations with many zeros, using statistical models to predict crash frequency seems not to be accurate for such crash datasets^26^. Decision Trees (DTs) and ensemble algorithms related to DT, such as RF and boosting algorithms, which are explained in the following subsections, are more interpretable compared to other ML algorithms and seem to be more appropriate for model development^9,10^.

#### Decision tree (DT)

DT model is a common supervised learning model and decision support tool for classification. This model classifies the data by learning simple decision rules derived from the data features. The maximum depths of the tree and minimum sample split are the parameters that need to be determined in the calibration process^8,10^.

In recent years, non-parametric techniques have become popular and have been used in traffic crash severity modelling, including Classification and Regression Trees (CARTs) to estimate crashes. CART is a simple but powerful approach to data analysis, and no predefined assumption is required to develop a CART model. In addition, while the correlation between explanatory and dependent variables is important in regression models, it is not a major concern in CART models^43^. Furthermore, CART models provide a graphical structure, including a tree with many branches and leaves for results. Graphical features assist in better understanding and interpreting the results^44^.

#### Random forest (RF)

Although DTs are non-parametric and low-biased, they suffer from a high variance, which makes them less useful for most practical applications. By aggregating multiple decision trees, one can reduce the model output variance significantly, thus improving performance. While this could be archived by simple tree bagging, the fact that each tree is built on a bootstrap sample of the same data gives a lower bound on the variance reduction due to the correlation between the individual trees. RF addresses this problem by sub-sampling features, thus de-correlating the trees to a certain extent, allowing for a more significant variance reduction and increasing performance^45^.

The RFs are an ensemble of de-correlated decision trees^44^, and this method combines bootstrapping and random feature selection. A bootstrapped tree grows with a randomly selected training sample from a training dataset, where the replacement is random, to determine the best variable/split-point in growing trees^18,24^. The RF can be applied for classifying data and classification problems based on the majority of the class votes in each tree. RF and totally randomized trees report feature importance as permutation accuracy (also known as mean decrease accuracy).

#### eXtreme gradient boosting (XGBoost)

eXtreme Gradient Boosting (XGBoost) is a decision tree-based ensemble ML algorithm that provides a gradient boosting framework. It is an iterative learning algorithm that weak ensemble learners in a unified model. Gradient boosting employs gradient descent to enable the optimisation of more complex differentiable loss functions. Rather than using the residuals of the previous model to train new models, gradient boosting uses the gradient of the loss function to train new models. Due to the use of second-order gradient descent, XGBoost is a very fast, efficient and accurate tree boosting algorithm^26^.

Xgboost and RF are similar models, not identical. RF uses a bagging ensemble model, while XGBoost uses boosting ensemble model, so it may sometimes differ in results. When the correlation between influential features is high, XGBoost will pick one feature and may use it while breaking down the tree further (if required), and it will ignore some/all the other remaining correlated features because using them will prevent learning new aspects of the model as they are highly correlated with the selected feature. But in Random Forest, the tree is not built from specific features, rather there is a random selection of features (by using row sampling and column sampling), and then the model as a whole learns different correlations of different features^45–47^.

### Model evaluation

Many measures exist to evaluate the performance of a model in solving classification problems. Traditionally, accuracy, weighted average precision, weighted average recall, and weighted average F-Score (Eqs. 1–7) are among the most popular measures for evaluating classification DTs^48^.

**Figure 4:**
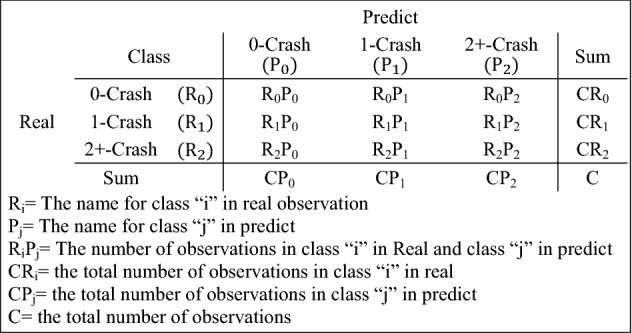
General confusion matrix for DT based models.

The following equations are based on the Fig. 4 variables which are presented in:

1 $$Accuracy = \frac{{\mathop \sum \nolimits_{i} \mathop \sum \nolimits_{j} R_{i} P_{j} }}{{\mathop \sum \nolimits_{i} CR_{i} }}\;\;\;\;i = j$$

2 $$Recall_{i} = \frac{RiPi}{{RiPi + \mathop \sum \nolimits_{i} \mathop \sum \nolimits_{j} R_{i} P_{j} }}\;\;\;\; i \ne j$$

3 $$Precision_{i} = \frac{RiPi}{{RiPi + \mathop \sum \nolimits_{i} \mathop \sum \nolimits_{j} R_{j} P_{i} }}\;\;\;\; i \ne j$$

4 $$F - Score_{i} = \frac{{2 \times \left( {Recall_{i} + Precision_{i} } \right)}}{{Recall_{i} + Precision_{i} }}$$

5 $${\text{Weighted Average Recall}} = {\raise0.7ex\hbox{${\mathop \sum \nolimits_{i} CR_{i} \times {\text{Recall }}_{i} }$} \!\mathord{\left/ {\vphantom {{\mathop \sum \nolimits_{i} CR_{i} \times {\text{Recall }}_{i} } C}}\right.\kern-\nulldelimiterspace} \!\lower0.7ex\hbox{$C$}}$$

6 $${\text{Weighted Average Precision}} = {\raise0.7ex\hbox{${\mathop \sum \nolimits_{i} CR_{i} \times Precision_{i} }$} \!\mathord{\left/ {\vphantom {{\mathop \sum \nolimits_{i} CR_{i} \times Precision_{i} } C}}\right.\kern-\nulldelimiterspace} \!\lower0.7ex\hbox{$C$}}$$

7 $${\text{Weighted Average F - Score}} = {\raise0.7ex\hbox{${\mathop \sum \nolimits_{i} CR_{i} \times F - Score_{i} }$} \!\mathord{\left/ {\vphantom {{\mathop \sum \nolimits_{i} CR_{i} \times F - Score_{i} } C}}\right.\kern-\nulldelimiterspace} \!\lower0.7ex\hbox{$C$}}$$

Since this study aims to identify the rural network's high-risk locations in terms of crash frequency, some evaluating measures based on excited measures are defined in Table 4 to evaluate the model performance. The measures are based on terminologies defined in a sample confusion matrix in Fig. 4. Predicting medium segment-day as a low-risk segment-day or predicting high-risk segment-day as a medium or low-risk segment-day is undesirable and dangerous in traffic safety planning while predicting low-risk segment-day as medium or high-risk segment-day is not undesirable.

**Table 4:** Evaluating measures computed from elements of confusion matrix to compare the accuracy of crash frequency prediction models.

No	Name	Description	Formula
1	TP0	True predict as 0 Crash	$\frac{{R_{0} P_{0} }}{{CR_{0} }}$
2	TP1	True predict as 1 Crash	$\frac{{R_{1} P_{1} }}{{CR_{1} }}$
3	TP2+	True predict as 2+ Crashes	$\frac{{R_{2} P_{2} }}{{CR_{2} }}$
4	FP0	False predict as 0 Crash	$\frac{{R_{1} P_{0} + R_{2} P_{0} }}{{CR_{1} + CR_{2} }}$
5	TP1,2+	True predict as 1 and 2+ Crashes	$\frac{{R_{1} P_{1} + R_{2} P_{2} }}{{CR_{1} + CR_{2} }}$
6	TPC	True predict as crash occurrence	$\frac{{R_{1} P_{1} + R_{1} P_{2} + R_{2} P_{1} + R_{2} P_{2} }}{{CR_{1} + CR_{2} }}$
7	FP01	False predict as 0 and 1 crash	$\frac{{R_{1} P_{0} + R_{2} P_{0} + R_{2} P_{1} }}{{CR_{1} + CR_{2} }}$
8	FPC	False predict crash	$\frac{{R_{0} P_{1} + R_{0} P_{2} }}{{CR_{0} }}$
9	FP1,2+	False predict 1 and 2+ Crash	$\frac{{R_{0} P_{1} + R_{0} P_{2} + R_{1} P_{2} }}{{CR_{0} + CR_{1} }}$
10	FP2+	False predict 2+ Crash	$\frac{{R_{0} P_{2} + R_{1} P_{2} }}{{CR_{0} + CR_{1} }}$

The definitions of the measures are based on the following principles, which are also summarised in Table 4. Measures computed from elements of confusion matrix are used to evaluate the accuracy of the developed models based on correctly predicting the high-risk segments. Evaluation measures defined and weighted based on the following criteria.

- Correct prediction of high-risk segment-day, including classes of 1 and 2+ crashes, is more important than low-risk segment-day (0 class) prediction.
- Incorrect prediction of high-risk segment-day should be minimised.
- Incorrect prediction of low-risk segment-day is less important.

## Results and discussion

### Model implementation

All models in this study are developed using 80% (64% as the train data and 16% as the validation data) of the total dataset and tested on the remaining 20% of the total dataset. As mentioned before, SMOTE is used to overcome the imbalanced data issue and have a large number of zero segment-day. To be consistent with the literature^37^, SMOTE is applied only to the training data set, and the remaining data is kept similar to the observed data. Thus, validation and test datasets are not balanced in this paper. The DT, RF and XGBoost models are trained and tested using the same train, validation and test datasets.

**Figure 5:**
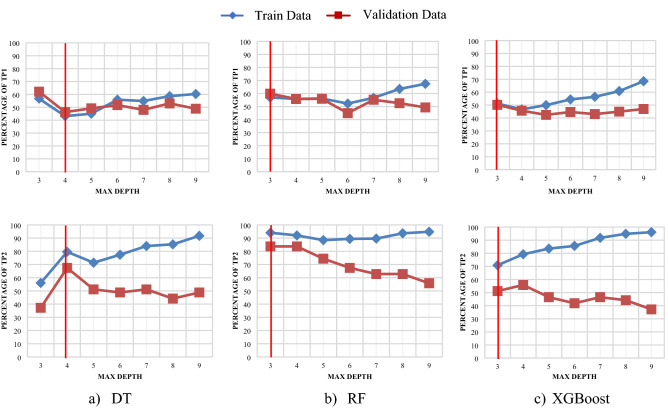
Maximum depth selection for different DT models.

In the model development procedure, one of the important steps is to select the hyperparameter (e.g. tree depth). Overfitting is often caused by allowing trees to grow deeper and deeper because more splits are made. Therefore, the model will fit perfectly for training data, but will struggle to generalise on test data. Underfitting will also occur if the depth is too low. Thus, determining the best max depth can combat overfitting and underfitting in the model. In this paper, the accuracy of classes 1 and 2+ curves in both training and validation procedure is used to determine the best depth for DT models as shown in Fig. 5. Generally, a high training accuracy indicates the higher learning capability of the models, while high validation accuracy shows the models’ generalisation capacity that can avoid the over-fitting of models. As shown in Fig. 5, the training data is shown in blue and the validation data is shown in red. The difference in models’ accuracy in training and validation data set will increase when the max depth goes beyond a threshold that indicates the model is being overfitted. To determine the max depth, select the curve point with the highest accuracy, in which the train accuracy curve grows with the increase in depth while the validation curve declines. The thresholds for DT, RF, and XGBoost are 4, 3, and 3.

The optimal DT hyper-parameters values include max depth: 4, min sample split: 4 and criterion: gini. The optimal RF hyper-parameters values include n_estimator: 2, max depth: 6, min sample split: 3, criterion: entropy, max feature: log2 and bootstarp: true. The optimal XGBoost hyper-parameters values include n_estimator: 2, max depth: 3, learning rate: 0.001, gamma: 0.9 and eval_metric: merror.

**Figure 6:**
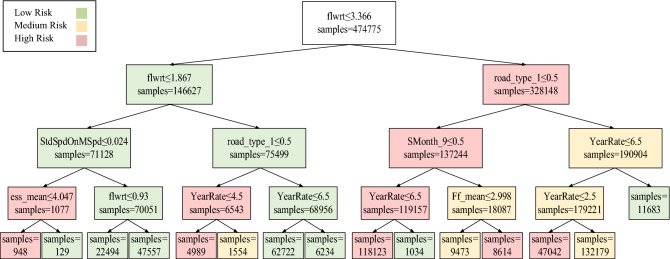
Result of DT with a maximum depth of 4.

The developed DT model is presented in Fig. 6. Probability distributions of crash frequency levels are estimated and assigned to each tree split by the selected important factors. As presented in Fig. 6, flow rate and road type are the main splitters in the DT. These two variables play an important role in classifying crash frequency prediction (more information is presented in "Model performance" section. and in Table 9). The CART method was applied for developing the DT. The model includes 29 nodes (15 terminal nodes), and the nodes are coloured according to their risk class (green: low-risk, yellow: medium-risk, red: high-risk).

### Model performance

The traditional evaluation measures for model comparison are presented in Table 5. As shown in this table, there is no model that outperforms in terms of all evaluation measures in all datasets. For instance, RF is the best model in the test and validation data set (Accuracy and Recall equal 0.70, Precision equal 0.97 and F-Score equal 0.81) and XGBoost is the best model in the train data set (Accuracy, Recall, Precision and F-Score equal 0.62). The XGBosst explains and interprets the factors affecting crash frequency, while the RF model is better for detecting trends and forecasting crash frequency. DT has a high score in accuracy, recall and F-score on the training dataset, the same as XGBoost, and in precision on the validation and test dataset, the same as RF. When these criteria are high, it indicates the correct diagnosis for each class of crashes.

**Table 5:** The Comparison of traditionally evaluation measures on calibration, validation, and test datasets.

Data Set	Model	Accuracy	Weighted average recall	Weighted average precision	Weighted average F-score
Train	DT	0.62	0.62	0.61	0.62
	RF	0.59	0.59	0.57	0.57
	XGBoost	0.62	0.62	0.62	0.62
Validation	DT	0.68	0.68	0.97	0.80
	RF	0.70	0.70	0.97	0.81
	XGBoost	0.67	0.67	0.67	0.67
Test	DT	0.68	0.68	0.97	0.80
	RF	0.70	0.70	0.97	0.81
	XGBoost	0.67	0.67	0.67	0.67

Table 6 presents the confusion matrix for all three models on the test data set. The confusion matrix is very important when models are applied to identify high-risk segments because, in a confusion matrix, the accuracy of prediction results is reported with the percent of correct prediction for each class. Since Class 1 and 2+ crashes are more valuable for identifying high-risk segments in comparison with 0-crash class, it is important to understand how the models are accurate in terms of predicting these two classes. As shown in this table, DT, RF, and XGBoost have the highest accuracy for classes 0, 1 and 2+ , respectively. Although, in terms of the main diagonal values of the confusion matrix, all three models have relatively the same performance, choosing the most accurate model based on the matrix is impossible unless weighting is considered for each element of the confusion matrix.

**Table 6:** Confusion matrix to present test results for all three models.

Observed classes	Predicted classes								
	DT			RF			XGBoost		
	0	1	2 +	0	1	2 +	0	1	2 +
0	68.48	25.60	5.91	68.26	25.62	6.12	67.62	27.67	4.72
1	32.08	45.24	22.68	31.61	41.25	27.14	31.49	48.41	20.09
2 +	12.96	48.15	38.89	12.96	33.33	53.70	12.96	51.85	35.19

To emphasise the importance of correctly identifying the high-risk segment-day, Table 7 presents the measures defined in Table 4 to compare the accuracy of the models. According to the results indicated in Table 7, none of the models considerably outperforms the others. The type of measures is positive and negative, which means the accuracy of the model increase when the measures increase for positive ones and decrease when the negative measures increase. Some of the defined measures are positive, which means the accuracy of the models increases with the increase in the value of the measures. Meanwhile, with negative measures by the decrease in the value of measures, the accuracy of the crash frequency prediction models increases. To measures an equal footing for comparing the accuracy of the developed models, all of them should be normalised. The methodology of the concordance analysis^48^ is used to normalise the measures using Eq. (8). The concordance analysis approach is used to establish the validity of a new diagnostic measuring or rating technique or to demonstrate the near-equivalence of multiple measuring or rating techniques^49^. The normalised measures are presented in columns 3 to 5 in Table 8. With the increase in the value of normalised measures, the accuracy of the models increases, and the decrease in those values means a decrease in the accuracy of the developed prediction models.

8 $$r_{ij} = \left\{ {\begin{array}{*{20}l} {\frac{{Z_{ij} }}{{max Z_{ij} }} \;\;\;\;if\, j\, is\, positive\, measure } \hfill \\ {1 - \frac{{Z_{ij} }}{{max Z_{ij} }} \;\;\;\;if\, j\, is\, negative\, measure } \hfill \\ \end{array} } \right.$$

where $r_{ij}$ is the value of normalized measures “j” for model “i” and $Z_{ij}$ is the value of measures “j” for model “i”.

**Table 7:** Evaluation summary for the three developed models.

No	Name	DT	RF	XGBoost	Type
1	TP0	68.48	68.26	67.62	Positive
2	TP1	45.24	41.25	48.41	Positive
3	TP2 +	38.89	53.70	35.19	Positive
4	FP0	30.39	30.50	30.38	Negative
5	TP1,2 +	44.86	41.99	47.62	Positive
6	TPC	69.06	69.50	69.61	Positive
7	FP01	33.81	32.49	33.48	Negative
8	FPC	31.52	31.74	32.38	Negative
9	FP1,2 +	31.37	31.66	32.17	Negative
10	FP2 +	6.19	6.48	4.98	Negative

**Table 8:** Normalising the final measures for model comparison and weight scenarios with the most accurate model in each scenario.

No.	Name	DT	RF	XGBoost	Senario1	Senario2		Senario3		Senario4	
					Equal weight	PF0C weight		PF weight		ACC weight	
1	TP0	1.00	1.00	0.99	0.10	0.08		0.08		0.15	
2	TP1	0.93	0.85	1.00	0.10	0.08		0.08		0.15	
3	TP2 +	0.72	1.00	0.66	0.10	0.08		0.08		0.15	
4	FP0	0.00	0.01	0.02	0.10	0.17		0.08		0.08	
5	TP1,2 +	0.94	0.88	1.00	0.10	0.08		0.08		0.08	
6	TPC	0.99	1.00	1.00	0.10	0.08		0.08		0.08	
7	FP01	0.00	0.04	0.01	0.10	0.17		0.08		0.08	
8	FPC	0.03	0.02	0.00	0.10	0.08		0.15		0.08	
9	FP1,2 +	0.03	0.02	0.00	0.10	0.08		0.15		0.08	
10	FP2 +	0.04	0.00	0.23	0.10	0.08		0.15		0.08	
Dominant model					XGBoost	XGBoost		DT		XGBoost	

The defined measures has different weights, and selecting the model with the highest accuracy is impossible without considering the measures' importance. To overcome this issue and have a valid comparison of the results from crash frequency prediction models, four weighting scenarios are defined in columns 6 to 9 in Table 8. In the first scenario, the weight of all the measures is assumed to be equal. In the second scenario, weights of FP0 (False Predict as 0 Crash) and FP01 (False Predict as 0 and 1 Crash), are assumed to be twice as important as the other measures. In this scenario, correctly identifying the segment-day with 1 and 2+ crashes is more important in terms of safety planning and management. Therefore, predicting the segment-day with 1 and 2+ crashes as segment-day with zero crashes and predicting the segment-day with 2+ crashes as segment-day with 1 crash have more weight in the second scenario. In the third scenario, weights of FP0, FP01 and FP2+ (False Predict as 2+ Crash) are twice as important as the other measures. In the fourth scenario, accurate prediction of true classes is important, and consequently, the defined weights of TP0 (True Predict as 0 Crash), TP1 (True Predict as 1 Crash) and TP2+ (True Predict as 2+ Crashes) are twice as important as the other measures.

The last row in Table 8 presents the dominant model for each weighting scenario. According to the results from Table 8, the DT model has high scores in ACC0, PFA and PFA01 which means the DT model is accurately predicting the segment-day placed in 0-crash class. RF model has the highest score in ACC2+ and PFNA01 and both measures show that RF model is accurately predicting the segment-day placed in 2+ -crash class. XGBoost model has the highest score in ACC1, PFNA, PTA1,2+ , PTA and PFA2+ which show XGBoost model is accurate in correctly predicting different crash classes for different segment-day, particularly segment-day in 1-crash class.

As shown in Table 8, XGBoost model is the most accurate crash frequency prediction model based on concordance analysis in the first, second and fourth scenarios. In the third scenario, DT model is the most accurate crash frequency prediction model. Thus, XGBoost is the recommended model for identifying the high-risk segment-day.

### Variable importance

The variables’ importance varies across different models. However, some variables are found to be the most influencing variables in different model types. Findings from Table 9 present that certain groups of variables, such as “Flow Rate” in traffic variables, “Road Type1” in road variables, “Year” in Calendar variables and Wind Speed in Weather variables, are more influential. Feature importance for selected variables in three models is reported in Table 9.

**Table 9:** Feature importance of variables in developed models.

Categorical Data	Feature	DT	RF	XGBoost
Traffic	Flowrate	0.48	0.31	0.24
	VolOnCap	0.00	0.19	0.11
	StdSpdOnMSpd	0.02	0.00	0.00
	SpdOnFrSpd	0.00	0.01	0.04
	SpeedOnHeadway	0.00	0.01	0.00
Road geometric	road_type_1	0.23	0.19	0.34
	road_type_2	0.00	0.02	0.01
	line_count_3	0.00	0.08	0.00
Calendar	Year	0.21	0.17	0.12
	SMonth_7	0.00	0.02	0.00
	SMonth_9	0.05	0.00	0.08
Weather	Wind	0.01	0.00	0.02
	Snow	0.00	0.00	0.00
	LengthSunlight	0.00	0.00	0.05

According to Table 9, the flow rate is among the top two traffic variables in all models. Volume over capacity ratio and the ratio of speed and free-flow speed are less significant. However, they are ranked as influencing variables in RF and XGBoost models. The ratio of standard deviation over mean speed is significant in the DT model and speed over headway is significant in RF. Compared to previous studies, the significance of these variables has been expected. However, previous studies mainly used Annual Average Daily Traffic (AADT) since this study has been conducted at the aggregate level, the mean speed and standard deviation of speed^18,28,31,32^.

Type road 1, which stands for the main road, is a significant road variable in all models. Type road 2 that shows freeway is a significant variable in RF and XGBoost, while the number of lanes is a significant variable in RF model. The year is among the top five significant variables in all models. The year of the crash is a significant variable in the crash frequency prediction model and safety management plan since the sociocultural and governance changes over the years, and those chances influence traffic safety. According to Table 9, wind speed is a significant variable in the DT and XGBoost models, while sunlight duration per day is just significant in XGBoost model. Khorasan Razavi province is in the desert, and the increase in wind speeds and desert storms negatively impact vehicle movements and vehicle control by the driver and limits horizontal vision, which results in traffic crashes.

## Application

Crash Risk Maps are created based on detailed crash data, which captures the combined risk arising from the interaction between traffic parameters and the environment. Dynamic risk maps provide an objective view of the frequency of crashes in segment-day on road networks, used to identify high-risk, medium-risk, and low-risk segment-day as input to a safety management plan. Dynamic risk maps that show the safety condition in segments may change over time (different days) in some road segments, while they will not change in some other segments. The dynamic risk maps show that the safety risk in some segments is fixed during various days, and it may be due to some geometric problems in those segments, while safety risk changing over different days may be due to the changes in traffic conditions. Dynamic risk maps may assist decision-makers in finding appropriate solutions to solve safety concerns.

**Figure 7:**
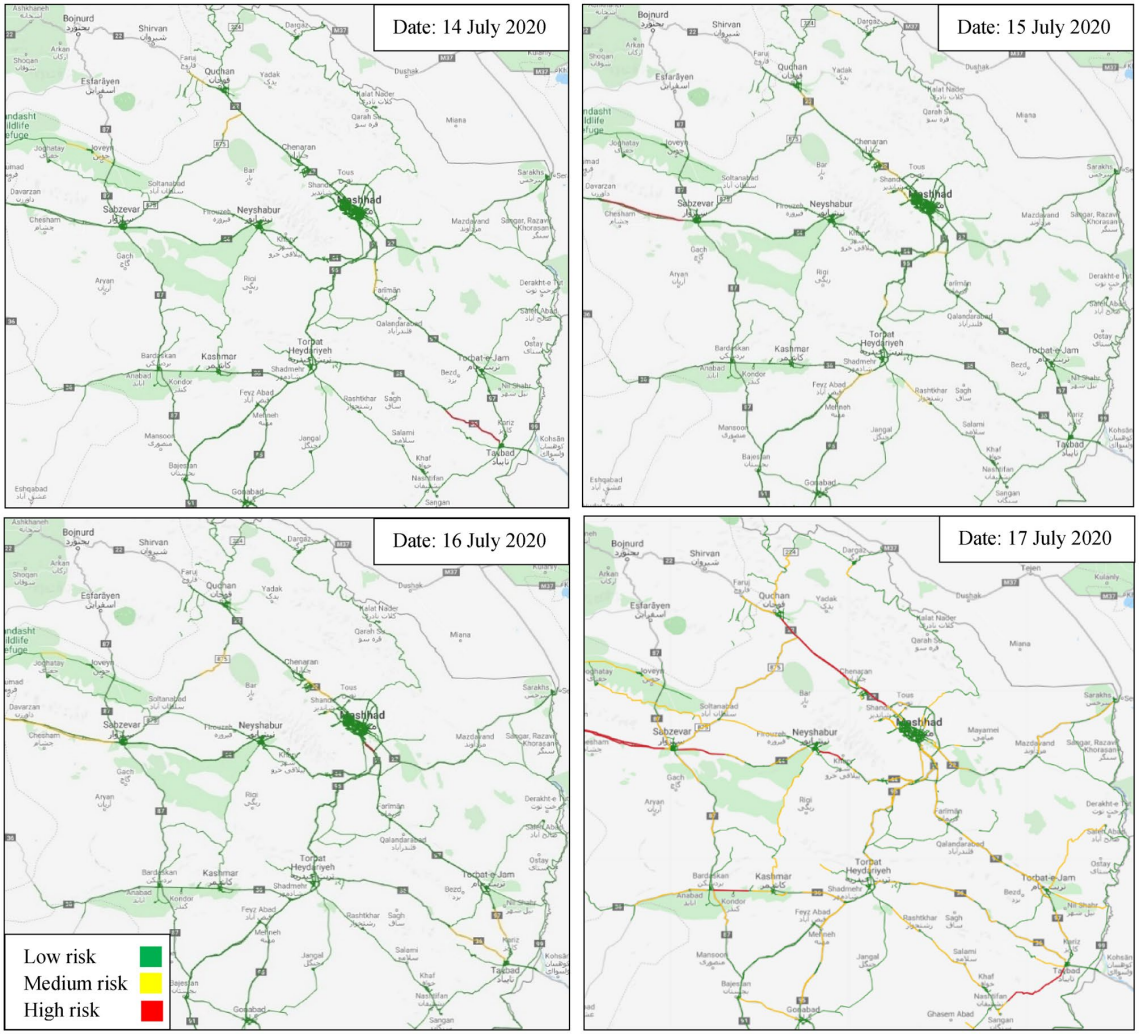
Risk map of the rural road network in Khorasan Razavi from 14 to 17 July 2020 [Maps Data: https://www.openstreetmap.org, Modified Map: Python 3.8.12].

The crash frequency model is paid for within a radius of 1500 m of loop detectors that assign the effects of each loop detector to a part of the road upstream and downstream. Figure 7 shows the spatial–temporal risk maps of a rural road network in Khorasan Razavi from 14 to 17 July 2020 based on the XGBoost model forecast, which indicate how the dynamic risk map change on different days and segments. Similar maps can be produced employing the proposed method for every day and every location in the rural road network. However, the crash risk maps for four days for all detectors have been shown as an example in Fig. 7. According to the figures, the crash risk will change over four days which means that a specific segment may be high-risk on some days but low-risk on others. Such segments seem to be influenced by temporal variables. However, segments with similar crash risk levels over different days may be influenced by spatial variables. The crash frequency results have 70% accurate prediction for high and medium risk classes in road segment-day.

## Conclusions and future research directions

This paper analysed traffic crashes on rural roads to prioritise high-risk segments on different days in LMICs. The crash severity data of Khorasan Razavi province, the second most populated province in Iran, was used in this research. This paper applied tree-based models to predict crash frequency considering different influencing factors, including traffic parameters, road characteristics, weather conditions and the calendar date. This paper also ranked segments of the rural road network on different days using the developed crash frequency prediction models. A high-resolution data indicating the effects of the temporal and spatial variations were excavated using a DT, RF and XGBoost. The frequency of crashes was divided into three classes: low, medium and high-risk. The classes include 0-crash, 1-crash, and 2 and more crashes equivalent to low, medium and high-risk segments, respectively. The imbalance of the number of classes in the training data was then corrected using SMOTE method to increase the generalisation capability of the developed models.

Based on the traditional evaluation measures, the XGBoost explains and interprets the factors influencing crash frequency, while the RF model is more accurate in detecting trends and forecasting crash frequency. According to the analysis results, the most accurate model to predict high-risk segment-day is XGBoost, the most accurate model to predict segment-day with 2+ crashes is RF, and the most accurate model to predict low-risk segment-day is DT model. To emphasise the importance of correctly identifying the high-risk segment-day, ten measures, including TP0, TP1, TP2+ , FP0, TP1,2+ , TPC, FP01, FPC, FP1,2+ and FP2+ are defined using elements of confusion matrix. All measures are considered for comparing models in a multi-criteria decision-making method called concordance analysis. Finally, XGBoost model has been selected as the most accurate model in predicting high-risk segment-day.

The main purpose of risk maps is to assess the frequency of collisions in road network link segments and to identify high-risk, medium-risk, and low-risk segments to assist drivers, road managers, and safety experts in reducing the frequency of crashes. The risk map in this paper is created from traffic, meteorological, geometric and calendar variables, based on the model estimates. All data are available as a supplementary file. The model consists of three classes of crashes: class 0-crash, class 1-crash, and class-2+ crash, which are labelled as low, medium, and high-risk crashes, respectively. The developed models can be used to dynamically predict the then safety status of the rural road networks and provide drivers with real time safety information. Designing decision support systems that compare risk maps on different days to identify the influential countermeasure for safety management in rural roads can also be a future direction for this research.

Using a limited number of loop detectors on rural roads for traffic data detection is a limitation in current crash frequency analysis and prediction studies. A possible future research avenue is to fuse loop detector data with other data sources, such as camera data. Moreover, using data on geometric design (e.g. vertical and horizontal curves) can improve the accuracy of crash frequency prediction models (Supplemenatry Information 1, Supplemenatry Information 2).

## Supplementary Information

Supplementary Information 1.

Supplementary Information 2.

## Data Availability

The datasets generated during and/or analyzed during the current study are available from the corresponding author on reasonable request.
